# Role of Therapeutic Endoscopic Ultrasound in Management of Pancreatic Cancer: An Endoscopic Oncologist Perspective

**DOI:** 10.3390/cancers15123235

**Published:** 2023-06-18

**Authors:** Dushyant Singh Dahiya, Saurabh Chandan, Hassam Ali, Bhanu Siva Mohan Pinnam, Manesh Kumar Gangwani, Hashem Al Bunni, Andrew Canakis, Harishankar Gopakumar, Ishaan Vohra, Jay Bapaye, Mohammad Al-Haddad, Neil R. Sharma

**Affiliations:** 1 Division of Gastroenterology, Hepatology & Motility, The University of Kansas School of Medicine, Kansas City, KS 66160, USA; 2 Division of Gastroenterology and Hepatology, CHI Creighton University Medical Center, Omaha, NE 68131, USA; 3 Department of Internal Medicine, Brody School of Medicine, East Carolina University, Greenville, NC 27834, USA; 4 Department of Internal Medicine, John H. Stroger, Jr. Hospital of Cook County, Chicago, IL 60612, USA; 5 Department of Internal Medicine, The University of Toledo, Toledo, OH 43606, USA; 6 Department of Internal Medicine, Indiana University School of Medicine, Indianapolis, IN 46202, USA; 7 Division of Gastroenterology and Hepatology, University of Maryland School of Medicine, Baltimore, MD 21201, USA; 8 Department of Gastroenterology and Hepatology, University of Illinois College of Medicine at Peoria, Peoria, IL 61605, USA; 9 Department of Internal Medicine, Rochester General Hospital, Rochester, NY 14621, USA; 10 Division of Gastroenterology and Hepatology, Indiana University School of Medicine, Indianapolis, IN 46202, USA; 11 Interventional Oncology & Surgical Endoscopy Programs (IOSE), GI Oncology Tumor Site Team, Parkview Cancer Institute, Parkview Health, Fort Wayne, IN 46845, USA

**Keywords:** endoscopic ultrasound, pancreatic cancer, treatment, radiotherapy, ablation

## Abstract

**Simple Summary:**

Pancreatic cancer is a highly aggressive disease associated with poor clinical outcomes. It is the seventh leading cause of cancer-related death worldwide. Due to the lack of obvious signs and symptoms until advanced disease, establishing an early diagnosis is often difficult. EUS is the imaging modality of choice to establish a diagnosis of pancreatic cancer. However, over the years, EUS has evolved to have therapeutic applications in the management of pancreatic cancer. EUS-guided fine needle injection of anti-tumoral agents, EUS-guided radiotherapy, and EUS-guided ablation techniques have become vital tools for endoscopic oncologists, especially in the management of locally advanced pancreatic cancer. These EUS-guided techniques offer high efficacy and demonstrate an excellent safety profile. However, they have not yet become routine practice due to the lack of long-term efficacy outcomes. Hence, additional studies are needed to investigate the long-term clinical outcomes and establish standardized procedural protocols for these techniques.

**Abstract:**

Pancreatic cancer is a highly lethal disease with an aggressive clinical course. Patients with pancreatic cancer are usually asymptomatic until significant progression of their disease. Additionally, there are no effective screening guidelines for pancreatic cancer in the general population. This leads to a delay in diagnosis and treatment, resulting in poor clinical outcomes and low survival rates. Endoscopic Ultrasound (EUS) is an indispensable tool for the diagnosis and staging of pancreatic cancer. In the modern era, with exponential advancements in technology and device innovation, EUS is also being increasingly used in a variety of therapeutic interventions. In the context of pancreatic cancer where therapies are limited due to the advanced stage of the disease at diagnosis, EUS-guided interventions offer new and innovative options. Moreover, due to their minimally invasive nature and ability to provide real-time images for tumor localization and therapy, they are associated with fewer complication rates compared to conventional open and laparoscopic approaches. In this article, we detail the most current and important therapeutic applications of EUS for pancreatic cancer, namely EUS-guided Fine Needle Injections, EUS-guided Radiotherapy, and EUS-guided Ablations. Furthermore, we also discuss the feasibility and safety profile of each intervention in patients with pancreatic cancer to provide gastrointestinal medical oncologists, radiation and surgical oncologists, and therapeutic endoscopists with valuable information to facilitate patient discussions and aid in the complex decision-making process.

## 1. Introduction

Pancreatic cancer has been identified as the 12^th^ most common cancer and the seventh leading cause of cancer-related mortality worldwide [1]. Pancreatic ductal adenocarcinoma (PDAC) is the most common subtype of pancreatic cancer, while other slow-growing pancreatic cancers include neuroendocrine tumors and pancreatic exocrine cancers [2]. The median age at diagnosis of pancreatic cancer is 70 years, but it is rarely seen in individuals below the age of 55 years [3]. Risk factors implicated in the development of pancreatic cancer include family history, genetic syndromes, smoking, chronic pancreatitis, increasing age, male sex, obesity, diabetes mellites, a diet high in fats and meats, African American race, and occupational exposures, among others [2,4]. Despite significant advancements in our knowledge and understanding of pancreatic cancer, it continues to be a lethal disease with a highly aggressive clinical course [5].

Most patients with early-stage pancreatic cancer lack obvious clinical signs and symptoms [6]. However, symptoms become apparent as the disease advances and starts to invade local tissues or metastasizes to distant organs [4]. Typical presenting symptoms of pancreatic cancer include abdominal or mid-back pain, obstructive jaundice, loss of appetite, maldigestion, unintentional weight loss, and cachexia [7]. Occasionally, complete pancreatic duct obstruction may also lead to recurrent bouts of acute pancreatitis [4,7]. With more advanced disease, patients may develop gastric outlet obstruction and venous thromboembolism (4). Traditional cross-sectional imaging techniques such as computer tomography (CT) and magnetic resonance imaging (MRI) may not be able to detect small or pre-malignant pancreatic lesions [8]. Therefore, an early diagnosis is often difficult to establish. Hence, these patients frequently present with advanced-stage disease or widespread metastasis leading to poor clinical outcomes and high mortality rates [6,9,10]. 

Endoscopic Ultrasound (EUS) is the imaging modality of choice for pancreatic cancer [11]. It is preferred over conventional CT and MRI due to a higher diagnostic yield and negative predictive value [12]. EUS may also be able to detect small pancreatic lesions which are often missed in cross-sectional imagining. Once the lesion is identified, an EUS-guided fine-needle biopsy (FNB) can be performed to confirm the diagnosis [13]. EUS-FNB has a specificity and sensitivity greater than 90% in the detection of pancreatic cancers [14,15]. Furthermore, a recent combination of EUS with artificial intelligence (AI) assisted models may also help in the early detection of pancreatic cancers and differentiate it from chronic and autoimmune pancreatitis with a high degree of accuracy [6,16,17,18]. However, despite its many advantages, EUS is not always available due to the technical complexity and the operator-dependent nature of the procedure, a steep learning curve for therapeutic endoscopists, and hospital limitations such as the need for specific equipment, specialized training of ancillary staff, and hospital costs [6,19]. 

The treatment of pancreatic cancer is highly dependent and guided by the stage at diagnosis [20]. In the early localized stage of the disease, surgical resection with adjuvant chemotherapy is the standard of care [21,22]. Recently there has been a shift towards a neoadjuvant approach with the hopes of reducing the risk of recurrence. In patients with “locally advanced” (>180-degree superior mesenteric artery or celiac artery involvement prohibiting resection) and late metastasized disease, the focus shifts to palliative chemotherapy, radiation therapy, and symptomatic management [23,24]. With the evolution of therapeutic endoscopy, EUS has become an integral part of pancreatic cancer management. Although the role of EUS in the diagnosis and staging of pancreatic cancer is well known, its utilization for the treatment of pancreatic cancer is infrequently discussed outside the realm of therapeutic endoscopy [24,25]. Hence, in this narrative review, we provide a detailed overview of the areas of utilization of EUS in the management of pancreatic cancer.

## 2. Discussion

Minimally invasive therapeutic endoscopic procedures have revolutionized the diagnosis and management of pancreatic cancers [6,26]. Compared to conventional surgical interventions, their minimally invasive nature, lower risk of complications, and significant improvement in the patient’s quality of life make them the preferred intervention in a select subgroup of patients [27]. The major applications of EUS in the management of pancreatic cancer are discussed below. 

### 2.1. EUS-Guided Fine Needle Injections of Anti-Tumor Agents

Although pancreatic cancers have a highly aggressive clinical course, exponentially increasing knowledge about tumor characteristics and microenvironments has led to significant advancements in medical therapy. However, an immune-suppressive microenvironment, the pathological hallmark of pancreatic cancer, hinders the efficacy of traditional chemotherapeutic agents and radiotherapy. This promotes tumor growth and eventually distant metastasis. Additionally, chemotherapy and radiation are associated with numerous systemic side effects and potential complications, thereby limiting utilization in all pancreatic cancer patients. As many patients may have locally advanced disease, EUS-guided fine needle injection (EUS-FNI) serves as a vital tool in the management of these complex patients. Using EUS-FNI, therapeutic endoscopists can easily gain direct focal access to the tumor within the pancreas or surrounding tissue, thereby minimizing systemic side effects and complications that are associated with anti-tumor agents. 

#### 2.1.1. Cytoimplant

In current literature, numerous studies have investigated the combination of EUS-FNI with different anti-tumor therapeutic agents. In 2000, the Phase I clinical trial by Chang et al. evaluated the efficacy and safety of cytoimplant (allogenic mixed lymphocyte culture) delivered via EUS-FNI in eight patients with advanced unresectable PDAC [28]. The cytoimplant induces the release of cytokines which activate various effector immune mechanisms leading to PDAC regression [29]. After therapy, the authors noted a partial response in two patients and a minor response in one patient with a median survival time of 13.2 months (28). There were no procedure-related complications [28]. However, in the last decade, there have been no other active clinical trials for cytoimplants despite somewhat promising results [29]. 

#### 2.1.2. Dendritic Cell-Based Immunotherapy

The combination of Dendritic Cell (DC)-based immunotherapy and EUS-FNI has also yielded positive results. DCs, one of the most potent antigen-presenting cells, works by activating T-cells and Natural Killer (NK) cells to directly destroy the pancreatic cancer tumor cells [30]. In a pilot study by Irisawa et al., EUS-FNI was performed to directly inject immature DCs intratumorally in seven patients with advanced PDAC who had previously failed chemotherapy with gemcitabine [31]. The authors noted that the intratumor injection via EUS-FNI was highly safe and effective with no clinical toxicity or intraprocedural complications [31]. Additionally, despite the fact that these patients had previously failed gemcitabine therapy, there was some clinical response with a median survival time of 9.9 months [31]. A later study by Hirooka et al. in 2009 utilized a combination of intravenous gemcitabine, EUS-FNI of OK432-pulsed DCs intratumorally, and subsequent intravenous lymphokine-activated killer cell infusions at 2-week intervals in five patients with locally advanced pancreatic cancer unamenable to surgical resection [32]. No treatment-related and intraprocedural adverse events were observed by the authors [32]. Of the five patients, three had an effective response to treatment with one partial remission and two with stable disease for more than 6 months [32]. Based on this background, Hirooka et al. conducted another Phase I trial from 2007–2015 to study the efficacy and safety of combined EUS-guided intratumoral injection of zoledronate-pulsed DCs (Zol-DCs), gemcitabine and αβT cells in 15 patients with locally advanced PDAC [33]. The authors reported that all patients completed the treatment, of which, seven had stable disease and most showed a long-term clinical response with a median overall survival time of 15 months [33]. There were no procedure-related adverse events [33]. In recent years, a novel approach of EUS-FNI delivered DC-based immunotherapy (neoadjuvant therapy) prior to surgical intervention for pancreatic cancer has also been studied [34]. Preoperatively, Endo et al. enrolled nine patients in the trial group that received EUS-FNI of OK432-pulsed immature DCs, while 15 patients in the control group did not [34]. Overall, the authors reported no severe systemic toxicities and intraprocedural complications of EUS-FNI [34]. Furthermore, two patients who received immature DCs, one of whom had stage IV pancreatic cancer with distant nodal metastasis, survived more than 5 years without additional adjuvant therapy [34]. Ultimately, based on the results, the authors concluded that preoperative EUS-FNI was feasible and safe as neoadjuvant therapy prior to surgical intervention in patients with pancreatic cancer [34]. 

#### 2.1.3. Oncolytic Viruses

The utilization of intratumor EUS-FNI-delivered oncolytic viruses (adenovirus, herpesvirus, and reovirus) has gained traction in recent years for pancreatic cancers [35]. Oncolytic viruses penetrate the tumor microenvironment and enter the tumor cells [35,36]. Thorough multifactorial mechanisms such as rapid uninhibited viral replication, production of pro-cytotoxic proteins, and activation of anti-tumor immunity, oncolytic viruses cause cell lysis and tumor destruction [36,37]. A Phase I/II clinical trial was conducted to assess the efficacy and safety of ONYX-015 (E1B-55kD gene-deleted replication-selective adenovirus) delivered intratumorally in eight sessions via EUS-FNI in 21 patients with unresectable PDAC [38]. Intravenous gemcitabine was also administered in combination with ONYX-015 during the last four sessions in these patients [38]. The authors observed that two patients each had partial regression and minor response to treatment, while six had stable disease [38]. The median survival time was 7.5 months [38]. Due to treatment toxicity or progression of pancreatic cancer, 11 patients had to discontinue the treatment [38]. Sepsis was noted in two patients before prophylactic antibiotics could be administered [38]. From a procedural standpoint, two patients developed duodenal perforation; however, when the EUS approach was changed from transduodenal to transgastric, no perforations occurred [38]. The authors concluded that transgastric ONYX-015 injection intratumorally via EUS with or without gemcitabine was relatively safe and feasible for the management of patients with unresectable PDAC [38]. Another Phase I clinical trial investigated the safety and effectiveness of EUS-FNI delivered HF10, a mutated oncolytic virus derived from herpes simplex virus-1, in combination with erlotinib and gemcitabine for 12 patients with locally advanced unresectable PDAC [39]. Of the nine patients who completed treatment, three were partial responders, four developed stable disease, and two had progressive disease [39]. The overall median survival time was noted to be 15.5 months [39]. Although one patient developed perforative peritonitis following duodenal stenosis, this was unrelated to the utilization of EUS-FNI [39]. Most recently in 2020, a Phase I clinical trial by Lee et al. assessed the utilization of intratumoral EUS-FNI of a combination therapy of Ad5-DS (adenovirus-mediated double-suicide gene therapy), oral 5-fluorocytosine, valganciclovir, and standard dose of intravenous gemcitabine in 11 patients with locally advanced PDAC [40]. As the study progressed, only nine patients were able to complete the treatment and two withdrew consent [40]. At 12 weeks, eight patients had stable disease and one had a partial response, but two patients experienced disease progression at 6.5 months [40]. The median progression-free survival was 11.4 months [40]. No procedure-related adverse events or complications were reported by the authors [40].

Tumor necrosis factor-α (TNF-α) has been identified as a potential prognostic and therapeutic target in PDAC [41]. Hence, TNFerade^TM^ Biologic, a replication-deficit adenovirus vector carrying the human TNF gene, was developed for intratumoral delivery under EUS guidance [42]. The vector consists of a radiation-inducible early growth factor-1 (EGR-1) gene promoter that plays a key role in the expression and secretion of TNF-α [43]. TNF-α has a synergistic effect with chemotherapy and radiotherapy resulting in tumor lysis [42,44]. A Phase I/II non-randomized multi-centered study investigated the feasibility and safety of TNFerade^TM^ in conjunction with systemic 5-Florouracil chemotherapy and radiation therapy in 50 patients with locally advanced pancreatic cancer [45]. TNFerade^TM^ was injected intratumorally by EUS-FNI in 27 patients, and a percutaneous approach under CT or ultrasound (US) guidance was used in 23 patients [45]. The authors noted a complete response in one patient, while three had a partial response, four had a minor response, and 12 patients had stable disease, with a median overall survival rate of 297 days [45]. Furthermore, seven patients eventually had surgical resection due to tumor downstaging after treatment, of whom six had negative surgical margins [45]. Although gastrointestinal (GI) toxicities included GI bleeding, pancreatitis, and cholangitis, there were no intraprocedural complications due to EUS-FNI [45]. Building on this study, Herman et al. conducted a Phase III randomized multi-centered study that enrolled 304 patients with locally advanced PDAC to compare the standard of care (SOC) plus intratumoral TNFerade^TM^ therapy with SOC alone [46]. SOC consisted of radiotherapy with concurrent intravenous 5-Fluorouracil followed by gemcitabine or gemcitabine plus erlotinib maintenance therapy [46]. TNFerade^TM^ was injected intratumorally via EUS-FNI or CT/US-guided transabdominal percutaneous approach [46]. The authors did not find a statistical difference in the median survival and median progression-free survival between the two groups [46]. No intraprocedural or post-procedural adverse events were reported [46]. However, a multivariate regression analysis demonstrated that EUS-FNI of TNFerade^TM^ was a risk factor for lower progression-free survival compared to CT/ultrasound-guided transabdominal percutaneous approach [46]. Ultimately, the study showed that TNFerade^TM^ therapy is safe for PDAC but may not have any effect on overall survival rates [46]. Therefore, additional prospective multi-center studies are needed to further validate the efficacy of TNFerade^TM^ for PDAC.

#### 2.1.4. DNA Plasmids

The H-19 gene, mapped at the short arm of chromosome 11, is expressed in numerous cancers, especially PDAC [47]. It regulates oncogenes, tumor suppressor genes, and angiogenesis factors such as tumor necrosis factor-*α*, thereby playing an important role in tumor proliferation, migration and distant metastasis [48,49]. DNA plasmids targeting the overexpression of the genes under the control of H19 regulatory sequences have been studied in the context of PDAC [47,50]. In 2012, a Phase 1/2a clinical trial that enrolled nine patients with unresectable, locally advanced PDAC, aimed to study the efficacy and safety of EUS and Computer Tomography (CT)-guided intratumoral injection of BC-819, a DNA plasmid targeting the diphtheria-toxin gene on H-19 [50]. In this study, three patients received CT-guided injections and six patients received EUS-FNI of BC-819 [50]. Of the nine patients that received treatment, three had a partial response and two were downstaged and subsequently underwent surgical resection [50]. The authors reported no adverse events from the procedure and concluded that BC-819 can be safely administered intratumorally via EUS-FNI or CT-guided injection [50]. 

#### 2.1.5. Chemotherapeutic Agents

Chemotherapeutic agents form a vital component of standard therapeutic regimens for both early and late-stage pancreatic cancers [51]. However, they have not been thoroughly investigated in the realm of EUS-guided intratumoral injections. In a porcine animal study, Matthes et al. investigated the use of EUS-FNI for the delivery of OncGel (Macromed Inc.) to the porcine pancreas [52]. OncGel consists of Paclitaxel bound to a thermosensitive gel carrier [52]. After EUS-FNI, OncGel provided sustained localized concentrations of Paclitaxel in the porcine pancreas [52]. All animals tolerated the procedure well without any intraprocedural complications [52]. Hence, the authors concluded that EUS-FNI of OncGel may be a potential therapeutic option for unresectable PDAC in humans [52]. 

In 2017, a single-center retrospective study aimed to investigate the safety and feasibility of intratumoral EUS-FNI of Gemcitabine in patients with locally advanced and metastatic pancreatic cancer prior to the administration of conventional therapy [53]. A total of 36 patients with pancreatic cancer (3 stage II, 20 Stage III, and 13 stage IV) were enrolled in the study, all of whom received EUS-FNI of a median volume of 2.5 mL Gemcitabine in the concentration of 38 mg/mL [53]. The median overall survival time was noted to be 10.4 months, and four patients (20%) were downstaged and underwent R0 surgical resection [53]. No intraprocedural adverse events were reported by the authors reflecting an excellent safety profile of EUS-FNI [53].

**Figure 1:**
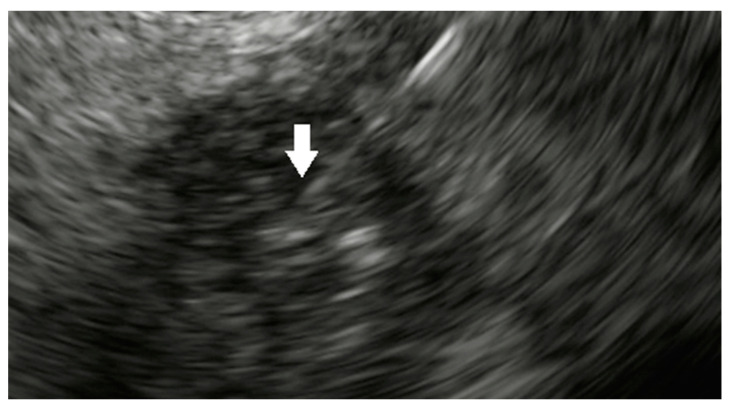
Administration of NanoPac^®^ into pancreatic cancer (white arrow).

Additional novel agents are currently being created and tested in clinical trials. A prime example of a very progressive novel agent is NanoPac^®^ (clincaltrails.gov NCT03077685/https://clinicaltrials.gov/ct2/show/NCT03077685; Accessed on 10 May 2023) [54]. NanoPac^®^ is submicron particle Paclitaxel (SPP) which when injected intratumorally leads to high therapeutic levels of Paclitaxel at the tumor site [55]. It causes tumor apoptosis and necroptosis and also stimulates the innate and adaptive immune response for its anti-neoplastic effect [55]. In an open-label, dose-escalating, Phase IIa clinical trial, patients with locally advanced PDAC have been enrolled from four centers in sequential cohorts of NanoPac^®^ at a volume up to 20% of tumor volume (maximum injection volume of 5 mL/patient) [54]. Each cohort has three patients, starting at the lowest concentration and sequentially titrating up [54]. The patients are followed for 1 year after NanoPac^®^ administration to evaluate overall survival, progression-free survival, tumor makers, response to therapy, and safety of NanoPac^®^ [54]. Results from a single center look promising [56]. Of the 13 patients with locally advanced PDAC or unresectable tumors, six were downstaged after NanoPac^®^ therapy and were eligible for surgical resection [56]. Furthermore, five of these six patients eventually underwent surgery, and four (80%) had R0 resection with two having no viable residual tumor (Figure 1) [56].

Overall, EUS-FNI of anti-tumor agents have highly variable clinical outcomes and may not improve overall survival rates. However, most studies have reported the procedure to be safe with minimal complications. Nonetheless, additional prospective, multi-center studies, with prior described and other novel agents, are needed to further investigate the efficacy of EUS-FNI of anti-tumor agents in patients with PDAC and to also develop standardized procedural techniques for therapeutic application of the procedure in the near future. 

### 2.2. EUS-Assisted Radiotherapy 

Compared to other solid tumors, pancreatic cancer is known to have higher rates of local recurrence and margin-positive resections [57]. Due to this high risk of recurrence, multimodal therapy consisting of a combination of surgical resection, chemotherapy, and radiotherapy (RT), is usually considered optimal treatment, particularly for patients with locally advanced disease [58]. External beam radiation therapy (EBRT), the most common method of radiation delivery, was the mainstay RT for PDAC in the past [59]. However, movements due to respiration, peristalsis, and random movements within the GI tract significantly limited the delivery of ablative doses of RT to the tumor site [58]. To overcome this challenge and ensure optimal delivery of RT to the tumor site, newer therapeutic interventions such as EUS-guided brachytherapy and stereotactic body radiotherapy (SBRt) after EUS-guided placement of a fiducial marker have emerged as viable treatment options [60,61]. 

#### 2.2.1. Brachytherapy

Brachytherapy involves the precise placement of radioactive seeds, microparticles or liquids which emit radiation directly into or next to the tumor site [62]. It allows for the direct local delivery of high doses of radiation to the tumor while minimizing damage to adjacent normal tissue [63]. Although numerous CT/US-guided techniques are available for brachytherapy, EUS-guided brachytherapy is the preferred method due to several advantages [64]. With EUS, therapeutic endoscopists are able to visualize real-time images for accurate positioning of the radioactive seeds [65]. Additionally, the puncture distance is shorter while using EUS compared to CT/US-guided techniques, and there is milder injury due to its minimally invasive nature [65].

In 2006, a pilot study enrolled 15 patients with unresectable PDAC (eight with stage III disease and seven with stage IV disease) to study the clinical response, safety and complications of EUS-guided interstitial brachytherapy without chemotherapy or external RT [66]. After a median follow-up of 10.6 months, 20%, 27% and 33% patients had minimal, partial, and stable disease, respectively [66]. Pain reduction was seen in 30% of the patients; however, it only lasted for a limited period of time [66]. Local GI complications included pancreatitis in three patients and pseudocyst formation in two patients, but there were no procedural complications [66]. Another prospective pilot study aimed to evaluate the efficacy and safety of EUS-guided interstitial brachytherapy with radioactive Iodine-125 seeds along with gemcitabine-based 5-fluorouracil chemotherapy in 22 patients with biopsy confirmed advanced pancreatic cancer [67]. The median follow-up and survival time were 9.3 months and 9 months, respectively [67]. Partial remission was noted in 13.6% of the cases, while 45.5% had stable disease [64]. There were no procedural complications [67]. However, PDAC ultimately progressed in 91% of the cases, all of whom died during the 2-year follow-up period [67]. The authors concluded that there was no survival benefit despite EUS-guided brachytherapy being a safe and viable treatment option for patients with advanced pancreatic cancer [67]. 

To further explore EUS-guided brachytherapy for locally advanced unresectable pancreatic cancer, an open-label, single-arm pilot study using phosphorus-32 (P-32) microparticles in combination with gemcitabine and nab-paclitaxel was devised in 2019. It is based on two previous phase II clinical trials which demonstrated an acceptable safety profile and tolerability of P-32 in combination with gemcitabine for locally advanced pancreatic cancer [68,69]. Although the study is currently ongoing, preliminary results from nine patients at three US sites demonstrated that the EUS-guided placement of P-32 was highly successful and technically feasible [68]. Furthermore, there were no procedure-related adverse events [68]. 

#### 2.2.2. Stereotactic Body Radiotherapy

SBRt is a relatively new innovative technique of precisely delivering high-dose external beam RT to extracranial tumor targets in a small number of sessions (fractions) [70]. It has been well-established in the treatment of numerous solid tumors such as non-small cell lung cancer, hepatic cancer, prostate cancer, and oligometastatic disease [71]. In recent years, SBRt has also garnered immense interest in the management of PDAC due to its excellent tolerability and shorter completion times (1-5 fractions), thereby taking less time away from conventional chemotherapy [72]. Furthermore, numerous studies have found SRBt to be comparable to EBRT in terms of survival outcomes and local tumor control [73,74,75].

Placement of a fiducial marker before SRBt helps with tumor localization and target tracking which helps to increase the overall accuracy of SRBt [76]. Traditionally, fiducial markers have been placed either intraoperatively or percutaneously under CT/US guidance. However, due to the availability and ease of placement, EUS-guided fiducial marker placement has gained popularity in recent years [61,77]. In 2006, a single-center prospective study that enrolled 13 patients with mediastinal and intra-abdominal tumors reported successful EUS-guided fiducial marker placement in seven (100%) patients with PDAC without any procedural complications [78]. Later in 2010, five studies investigated the safety and feasibility of EUS-guided fiducial marker placement in patients with PDAC [79,80,81,82,83]. A total of 133 patients with PDAC were included in these studies [79,80,81,82,83]. Of these patients, 121 (91%) had successful EUS-guided fiducial marker placement without complications, while 12 (9%) patients had a technical failure of the procedure [79,80,81,82,83]. Another single-center prospective study by Choi et al. which enrolled 32 patients, of which 29 had PDAC reported successful EUS-guided fiducial marker placement in all 29 (100%) patients [84]. However, one (3.4%) patient with PDAC developed mild pancreatitis requiring a prolonged hospitalization for 2 days after the procedure [84]. Dávila Fajardo et al. enrolled 23 patients with resectable, borderline resectable, or locally advanced PDAC for EUS-guided fiducial marker placement [85]. In total, 63 fiducial markers were placed [85]. The authors noted the technical difficulty in 11.3% (8 of 71 attempts) of fiducial marker placements and only one (4.3%) patient had a periprocedural adverse event in the form of minor bleeding [85]. Six days after the procedure, one (4.3%) patient presented to the hospital with cholangitis due to obstruction of the percutaneous transhepatic cholangiography (PTC) drain [85]. In 2016, Dhadham et al. conducted a large single-center retrospective study to evaluate the technical feasibility and safety of EUS-guided fiducial marker placement in 514 patients with numerous GI malignancies [86]. There were 188 patients with PDAC and a total of 510 EUS-guided fiducial markers were placed in these patients [86]. Technical success was achieved in 187 (99.5%) patients [86]. Post-procedural minor bleeding that resolved spontaneously was the only adverse event noted in seven (3.7%) patients with PDAC [86]. The authors concluded that EUS-guided fiducial marker placement should be adopted more broadly due to its feasibility and excellent safety profile [86]. The most recent single-center retrospective study by Tabernero et al. investigated the use of EUS-guided fiducial marker placement in 47 patients with biopsy-proven advanced PDAC [87]. The technical success rate of the procedure was noted to be 100% [87]. However, two (4.2%) patients developed adverse events including mild pancreatitis and duodenal abscess [87]. These findings aligned with previous studies that have demonstrated EUS-guided fiducial marker placement to be a highly safe and feasible procedure in the hands of experienced therapeutic endoscopists who are well-trained in EUS.

**Figure 2:**
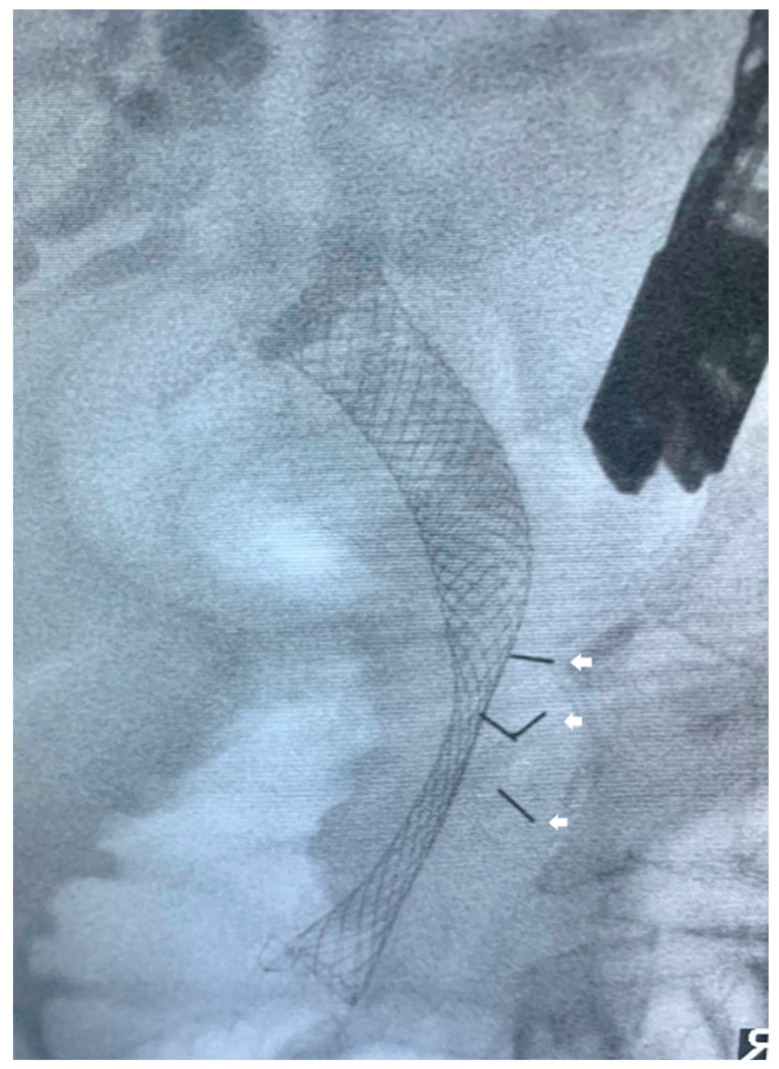
Metal stent in the bile duct with endoscopic ultrasound-guided Visicoil^TM^ fiducials (white arrow) placement in a patient with pancreatic head cancer for stereotactic body radiotherapy.

With recent technological advances in GI oncology, smaller and highly flexible fiducial markers have been introduced to circumvent the technical issues associated with traditional fiducial markers. Khashab et al. compared EUS-guided placement of the traditional fiducials (5-mm length, 0.8-mm diameter) with the newer Visicoil^TM^ fiducials (10-mm length, 0.35-mm diameter) in 39 patients with locally advanced pancreatic cancer [88]. The authors noted better visibility for traditional fiducials compared to Visicoil^TM^ fiducials without statistically significant differences in migration rates or technical difficulty [88]. Furthermore, no intra- or postprocedural complications were noted [88]. Hence, the authors advocated for the use of traditional fiducials over Visicoil^TM^ fiducials whenever possible [88]. Nonetheless, this study was important as it paves the way for potential improvements in the designs of fiducial markers to increase technical success rates and limit complications (Figure 2).

### 2.3. EUS-Guided Ablation 

#### 2.3.1. Radiofrequency Ablation

**Figure 3:**
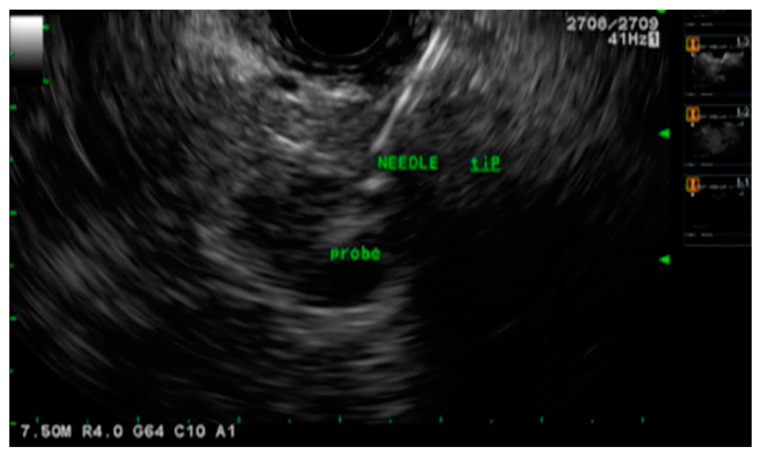
Endoscopic ultrasound-guided radiofrequency ablation.

Radiofrequency ablation (RFA) is a minimally invasive therapeutic intervention used to ablate neoplastic tissue by thermal coagulative necrosis [89]. It utilizes high-frequency alternating currents to generate temperatures ranging from 60–100 degrees Celsius which damages neoplastic cells and the tumor microenvironment [90]. Additionally, RFA also generates an intense immune response consisting of inflammatory B and T cells that are specific to the ablated tissue [91,92]. This further helps with tumor lysis. Although RFA is possible via open, laparoscopic, or percutaneous approach, EUS-guided RFA (EUS-RFA) is more advantageous as it is minimally invasive, has high efficacy, offers excellent tumor visualization and localization, provides real-time imaging guidance, and demonstrates an excellent safety profile (Figure 3) [61,93,94].

Animal studies using porcine models established EUS-RFA to be a highly safe and technically feasible procedure, thereby encouraging its use in humans [95,96,97]. In 2016, a single-center prospective study by Song et al. investigated the technical feasibility and safety of EUS-RFA in six patients with unresectable PDAC [98]. The procedure had a 100% technical success rate; however, two patients experienced mild abdominal pain after the procedure [98]. There were no other major adverse events such as GI bleeding, pancreatitis, duodenal injury, portal vein and/or splenic vein thrombosis, or procedure-related mortality [98]. Another single-center retrospective study assessed the feasibility, safety, and technical success of EUS-RFA in nine patients, of whom eight had locally advanced PDAC and one had pancreatic head metastasis from renal cell carcinoma [99]. After excluding one patient from the analysis due to the presence of a large necrotic area within the pancreatic tumor, the authors noted that EUS-RFA was feasible in 100% of the patients [99]. Three patients developed mild post-procedural abdominal pain which was managed conservatively [99]. Furthermore, there were no early or late major adverse events after the procedure, reflecting its excellent safety profile [99]. Most recently in 2018, Thosani et al. conducted a multi-center retrospective study from four centers in the US consisting of 21 patients that underwent EUS-RFA [100]. The most common lesion was PDAC seen in 10 (47.6%) patients [100]. The technical success rate of the procedure was noted to be 100% and only one patient had post-procedural complications of worsening abdominal pain [100]. The authors concluded that EUS-RFA is safe and technically feasible in the management of PDAC [100].

#### 2.3.2. Hybrid Cryothermal Ablation

A novel hybrid cryotherm probe (CTP) that combines RFA and cryogenic cooling under EUS guidance has also been tested for pancreatic cancer. Arcidiacono et al. investigated the utilization, feasibility, and safety of EUS-guided CTP (EUS-CPT) in 22 patients with locally advanced pancreatic cancer after neoadjuvant therapy [101]. The authors reported that EUS-CPT could be applied successfully in only 16 (72.8%) patients [101]. It was not possible in six (27.2%) patients due to the stiffness of the GI wall and the tumor [101]. No intraprocedural complications were noted, but three patients reported postprocedural abdominal pain with hyperamylasemia responsive to analgesics [101]. One patient developed post-procedural minor GI bleeding in the duodenal lumen, which was treated with endoscopic clip placement [101]. Late complications were related to tumor progression rather than the procedure [101]. Due to the small sample size of this study, firm conclusions could not be drawn, and the authors advocated for the need for additional studies to validate the findings [101].

#### 2.3.3. Microwave Ablation

Microwave ablation (MWA) technology is a new energy-based thermal ablation technique [102,103]. Microwaves are essentially electromagnetic waves with frequencies ranging from 900–2450 MHz [104]. When these waves interact with tumor tissue, they agitate water molecules and induce frictional heating of the tissue leading to cellular death via coagulative necrosis [102,104]. Compared to other ablative techniques, particularly RFA, MWA has several advantages such as the ability to reach higher intratumor temperatures, larger tumor ablation volumes, deeper penetration into tissues, faster ablation times, excellent maneuverability and optimal heating of tumors in close proximity of blood vessels, and less procedural pain [102,105]. Furthermore, EUS guidance for MWA provides therapeutic endoscopists with high-quality real-time imaging for precise therapy [106]. 

Although studies have investigated the use of MWA for pancreatic neuroendocrine tumors, its feasibility and safety in PDAC is still an area of active research. In animal studies using porcine models, EUS-guided MWA (EUS-MWA) was found to be safe and effective for pancreatic cancers without severe procedural complications [107]. Prospective and retrospective single-center studies have demonstrated MWA via percutaneous and open approaches to be feasible and safe for patients with locally advanced PDAC [108,109]. However, in the current literature, there are no large studies that have investigated the utilization, feasibility, and safety of EUS-MWA for PDAC. Nonetheless, EUS-MWA seems to be a promising prospect for the management of PDAC in the future.

#### 2.3.4. Photodynamic Therapy

Photodynamic therapy (PDT) is an innovative method of selective tissue necrosis using light, most frequently from lasers, after intravenous (IV) administration of a photosensitizing agent [110]. Due to their unique characteristics, there is a greater accumulation of photosensitizers in the neoplastic tissue compared to normal tissue [36]. The neoplastic tissue is then exposed to a specific wavelength of light under EUS-guidance which activates the drug to interact with oxygen, thereby generating singlet oxygen that causes localized tissue necrosis [111]. As a photochemical reaction is used in the process instead of a thermal one, the mechanical integrity of the surrounding normal tissue is maintained [112]. Furthermore, PDT uses non-ionizing radiation which mitigates the risk of radiation toxicity seen during conventional RFA [113].

Animal studies using porcine models have demonstrated that PDT can be performed successfully without severe complications [114,115]. In 2015, Choi et al. examined the safety and feasibility of EUS-guided PDT (EUS-PDT) for local tumor control in patients with locally advanced pancreaticobiliary malignancies [116]. Of the four patients enrolled in the study, only one (25%) had pancreatic cancer situated at the tail of the pancreas [116]. EUS-PDT was administered successfully in all patients [116]. The disease remained stable in all patients during a median follow-up of 5 months and there were no procedure-related complications [116]. Based on this background, an open-label, Phase I, single center, prospective study enrolled 12 patients with biopsy-proven treatment-naïve locally advanced pancreatic cancer to assess the safety and feasibility of EUS-PDT [117]. These patients received IV Porfimer Sodium (Concordia Laboratories Inc, St Michael, Barbados) followed by EUS-PTD 2 days later [117]. A CT scan was performed 18 days after EUS-PTD to assess pancreatic necrosis, and patients received IV Nab-paclitaxel and Gemcitabine 7 days after CT for 3–4 weeks [117]. The authors noted that there was an increase in the percentage of tumor necrosis in six (50%) patients after EUS-PTD compared to baseline imaging [117]. The median progression-free and overall survival were 2.6 months and 11.5 months, respectively [117]. Surgical resection was attempted in two patients, of whom one had a complete response [117]. Although there were eight serious events during the study, none were related to EUS-PTD [117]. Most recently in 2021, a pilot study by Hanada et al. enrolled eight patients with nonresectable locally advanced pancreatic cancer to assess the feasibility of EUS-guided verteporfin PDT [118]. Two days after EUS-PTD using verteporfin, five (62.5%) patients demonstrated a zone of necrosis on CT imaging, while three (37.5%) did not [118]. There were no intraprocedural or postprocedural adverse events, and no changes in patient-reported outcomes [118]. The authors concluded that EUS-guided verteporfin PDT is feasible for locally advanced pancreatic cancer, but patient enrollment and data collection are still ongoing for a Phase II clinical trial [118,119]. However, based on results available from current studies, EUS-FDT seems to be a safe and feasible salvage treatment option for patients with locally advanced pancreatic cancers who are poor surgical candidates and/or had progression of the disease despite conventional chemoradiotherapy. Table 1 summarizes EUS-guided ablation techniques for pancreatic cancer. 

## 3. Conclusions

Pancreatic cancer has an aggressive clinical course. Due to the lack of obvious clinical signs and symptoms, an early diagnosis is often difficult to establish. Hence, patients usually present with advanced disease at the time of diagnosis. EUS is the gold standard imaging modality of choice for pancreatic cancer due to its superiority over traditional cross-sectional imaging. Additionally, EUS-FNB can help establish a diagnosis of pancreatic cancer with a >90% specificity and sensitivity. In recent years, EUS has evolved from a purely diagnostic procedure to an important therapeutic intervention for the management of pancreatic cancers. EUS-FNI of anti-tumor agents, EUS-guided RT, and EUS-guided ablations have gained immense popularity due to their availability, feasibility, and excellent safety profile. Additionally, there is an interest in seeing the effects of these local therapies to propagate the body’s innate immune system and increase the response to immunotherapies or novel systemic agents. However, they have not yet become routine practice due to the lack of long-term efficacy outcomes. Given the knowledge gap that currently exists on these new approaches, continuous research on therapeutic EUS is warranted to not only investigate survival benefits but also establish standardized procedural protocols and help with device innovation to maximize efficacy while minimizing procedural complications. 

## Figures and Tables

**Table 1:** Endoscopic Ultrasound-guided ablation for pancreatic cancer.

Study	Year	Study Characteristics	Total Number of Patients	Technical Success (%)	Intraprocedural Complications	Postprocedural Complications
**Radiofrequency Ablation**						
Song et al. [98]	2016	Single Center Prospective	6 (unresectable PDAC)	100%	None	2 (mild abdominal pain)
Crinò et al. [99]	2018	Single Center Retrospective	9 (8 locally advanced PDAC, 1 pancreatic head metastasis)	100%	None	3 (mild abdominal pain)
Thosani et al. [100]	2018	Multi-center Retrospective	21 (10 PDAC)	100%	None	1 (abdominal pain)
**Hybrid Cryothermal Ablation**						
Arcidiacono et al. [101]	2012	Multi-center Prospective	22 (locally advanced PDAC)	72.8%	None	4 (3 abdominal pain with hyperamylasemia, 1 minor GI bleeding)
**Photodynamic Therapy**						
Choi et al. [116]	2015	Single Center Prospective	4 (1 pancreatic cancer)	100%	None	None
DeWitt et al. [117]	2018	Open-label Phase I Single Center Prospective	12 (locally advanced PDAC)	100%	None	None
Hanada et al. [118]	2021	Pilot Study	8 (locally advanced PDAC)	100%	None	None

PDAC: Pancreatic Ductal Adenocarcinoma. GI: Gastrointestinal.
